# The Secret Commission Evil

**Published:** 1911-04

**Authors:** J. P. Lord

**Affiliations:** Omaha


### THE SECRET COMMISSION EVIL.*

J. P. Lord, M. D., Omaha.

There seems to me to be a greater need to discuss one of the ethical questions agitating the surgical world to-day, than to add another scientific paper. It has seemed to me that specialism, with all the advantages and benefits which it has brought to mankind, enabling specialists to render such distinct service to humanity and thereby enhancing public confidence and esteem, has not been an unmixed blessing to the professional and readjustments in the relationships of patient, family physician and specialist, together with new economic conditions, as developed by our numerous public and private hospitals, dispensaries, laboratories, corporation work, indemnity insurance, lodge and society practice, have brought problems for solution by the profession in general, and our specialty in particular. The commercial spirit, with which the present age is now credited, has added another factor, the control of which we, with our somewhat neglected code of ethics, have not kept. The radical development of specialism, with its special rewards and the keen rivalry incident thereto, has developed a new order of things professionally, the ethical adjustments of which have not yet been generally made.

There has been so much said on this subject that it would seem puerile to say more. Indeed, it is not my purpose to try to improve on what has already been so well and fully presented. Realizing as I do that it is probably true that the practice of fee division, secret division, joint fees, commissions, so-called dichotomy, graft, and possibly obtaining money under false pretenses, is increasing, especially among the younger element, I undertake to arouse our members from their seeming lethargy. We teachers and hospital surgeons, as a rule, are established in our respective communities, and have not felt the force of this evil to so great a degree as to compel action. Those who follow will feel it keenly. Many young men are wrestling with this moral problem and will yield to the general trend of practice unless some aroused moral force within the profession, or the effect of publicity, checkmates this menace to professional honor, dignity and common honesty. I agree with those who favor publicity as the speediest and most effective remedy. Then, too, we need some Roosevelts to keep up a rapid-fire attack on this practice.

We should maintain a sentiment against any countenance of graft. Its devotees should not be given positions of honor. They should learn the penalty for unfair and unmanly competition. The present standards of membership of this society should prevail, and we would do well to revise our membership lists, since we have a considerable number who do business on a commercial basis. Our body is not in a position to reform the other fellows when.we, too are culpable. Scarcely a man among us is in a position to cast the first stone, but there are many who have recoiled from this thing on the discovery that their temptress had assailed their manhood and their integrity, and that hey had actually violated their patients' confidences.

In writing on this subject several years ago I made the statement that the division of fees was not itself so bad as that to which it leads. The road is short and direct to the practice of graft. This may be a semingly harsh term to apply to some of the most respectable forms of secret fee division. Secrecy, however, makes it dishonest and renders possible all forms of abuse. The results of the practice are so manifestly demoralizing as to cause all those who love their profession and cherish it sacred traditions, and who seek to uphold its honor and good name, to array themselves in opposition to those who seemed concerned only for the monetary rewards of to-day.

It has been observed that those practitioners who work on a commercial basis are prone to lose sight of the patient in their efforts better to serve their immediate selfish ends. Their lack of thorough consciousness leads them into questionable methods. They handle cases as merchandise instead of treating them as patients. This causes them to hold on too long before advising operation, and then to recommend operation on a moribund patient. Their zeal to have people operated on who are willing, causes them to work up cases, and this working-up process sometimes is so considerable that they claim compensation for it. The service is oftentimes considered by them of monetary value equal to or greate r than the skilled and experienced services renderd by the surgeon, who has possibly made or caused to have made various laboratory tests, and exhausted every resource to complete a diagnosis, and who has done the operating and given the after-care.

The specialists with their hospital connections and the valuables facilities for advanced and successful work, and the resulting wealthy and large clientele, cause the envious to consider that these men have the best of it, and they are willing to adopt grafting as their most ready means to easy money.

The practitioners who have for years adhered to an inelastic fee-bill find themselves with their financial wings clipped, and unable to rise above the level of customary fees. The specialist is somewhat of a law to himself and most often gets what he asks. These practitioners, therefore, avail themselves of the specialist's faculty of fee-getting, by requesting the specialist to add a sum for them, as the people would object to paying them a proper amount.

The specialists have often been accommodating in this way, and have saved themselves and the family physician the trouble of explaining the matter to the patient. The country doctor often plays "goody-goody," and says that he will not make a charge for accompanying the case if expenses are paid, of course depending on receiving a part of the specialist's fee. The specialist's fee is, therefore, under such circumstances, unnecessarily large. This gives the patient and all his friends an exaggerated idea of the value of the surgeon's services, and the relatively greater importance of the work of the specialist. The poor country doctor's services, therefore, become diminutive, judged from the cheapness of his fees. In other words, he is overshadowed and has cheapened himself.

Physicians of all classes must learn to make charges commensurate with the value of their services. Fee-bills hinder in this. The fee-bill is the union scale. It bolsters up the incompetent and often prevents the high-class, scientific man from getting his deserts. A hide-bound fee-bill and an increasing lack of appreciation of the practitioner's value, dwarf him, and compel him to resort to questionable methods for playing even. He must learn to elevate his standing by special fees for his improved methods of diagnosis and treatment. These have been acquired, perhaps, by special post-graduate courses, time abroad, and special equipment, and yet his price per visit or consultation is the same as it was twenty-five years ago.

Surgeon's fees may be no greater, but when we come to consider that it is possible for the surgeon to operate on several patients in the course of one morning, which is rendered possible by having the command of an unlimited hospital force, we can readily understand how the net results may be much greater than in the earlier days of surgery. On the other hand, the practitioner finds his work more exacting and painstaking, requiring more time, trouble and expense.

The rapid development of surgery during the last quarter century, with its greater rewards, has attracted so many to its ranks that competition for business has become so keen as to bring about these trade methods. Too many are now occupying the surgical field to be favored with teaching and hospital positions. Therefore, other means must be resorted to for attracting business. This custom of fee division, however, was probably established by some of the very men holding these places, and now their old weapon is turned against them.

Many of our surgeons throughout the country are ready-made after a few weeks at some of our large clinics, and then after self-styling themselves as surgeons and passing the word along the line that they are liberal and fair with the general practitioner, they are soon doing business and are not embarrassed by that period of waiting so well remembered by some of us.

Our medical schools have been remiss in their ethical instruction to students. The subject of fee division has been eschewed in some faculties for fear of engendering feeling, developing strife and endangering the integrity of the organization. The same science has prevailed in medical societies, because it might cost something to speak. Students are now without the close contact that was enjoyed in the days of the preceptor, whereas they now need more of the moral and ethical side than ever before.

Clearly so gross a violation of honesty and ethics, if continued, will lead to greater violations of fair play. If our tendency is to lower levels of conduct, it will be but a question of time before our noble profession will be out of favor with the people. There has never been a time when full confidence is more needed, becaus the co-operation and confidence of the people are necessary to secure the legislation needed for conserving the public health. This would aid the people in realizing our dreams of health and resultant happiness from the prevention of disease consequent on a universal crusade against violations of hygiene and health, and for the elimination of all preventable disease. A mercenary profession unworthy of public confidence will retard this movement and the unworthy members must be held to account by us and our standards maintained. At present the profession is becoming demoralized and our standards are being systematically assailed. Not only our standards, but those who stand for them, are attacked and their characters and motives are besmirched.

The graft element is going into medical politics and some county societies are controlled by this elemnt. The old guard is discredited and lowered standards are set up. The influence of these classes is against the American Medical Association, and their influence is soon to be felt in their organized opposition. The profession of Chicago and some other places do not have to go from home to read the handwriting on the wall.

The demoralization will be more complete when this practice extends to other lines than surgery. The movement has begun, and consultation practice and other specialties are now in cient standing to enable him to name a fancy price as the consultant's fees, which he collects and then remits the consultant the minimum fee. Any one of us can verify this by personal experience.

The primary object of fee-splitting is to get business. It is unfair competition. It has been my observation that fee-splitters are bad competitors in other ways. They will resort to other equally devious or questionable means of getting patients. They are usually underbidders; they lack sincerity; they are dishonest; they do not stop at dividing with physicians; hotel clerks, hack-men, news-agents, bar-kepers, ordinary clerks, traveling men, medical students, priests and preachers—all are represented, though few of the latter classes stoop to this practice. Those who do this will not stop at willful misrepresentation of men of high character and standing. Physicians go lower and lower who become infected with this vice, and their penchant for easy money becomes their undoing. Their methods become known in a communitly. Then they sell out, move to another locality, and imediately proceed to repeat their previous methods. These, of course, are exaggerated examples of the grafter, but a uphy-sician who grafts at all is not only unethical but alo dishonest.

The graft spirit has developed alarmingly within a decade, but a reaction has set in. Encouraging reports of progress have come from Chicago, Minneapolis and St. Paul, and I am pleased to report that a stand has been taken by the faculty of the University of Nebraska and of the Creighton Medical College. Whole states are, however, reported in the grasp of the system. Kansas, Iowa and Missouri, as well as many other states, are affected by it. From Kansas specialists reports that division of fees is too mild a term; that the surgeon must now accept what the general practitioner is willing to give him.

Hospital service is now required for graduation by some medical colleges and considerable numbers who have had hospital training and are qualified to do surgery go to our smaller cities and larger towns. To gain a foothold, notwithstanding the prestige of college professors and the large denominational city hospitals to meet competition, a resort to fee division and ultimate graft is the easiest course. The wail is going up in every quarter from our less enterprising but more conscientious confreres, and we are now hearing from some of our city brethren, wh oare beginning to observe the rather alarming prevalence of these methods of finance, exploitation of cases, and the merchandising of patients.

After all of this lugubrious, pessimistic and despairing talk of conditions in our profession I am still hopeful. Physicians as a class are the best people on earth, made so by their education, environment, every-day training in charity and human sympathy.

This evil thing had a very innocent and insidious start. The surgeon wanted to be fair and divided fees that the physician had in part actually earned. Nobody was harmed, for the pa-eitnt had not been wronged. This seemed innocent enough. Then came the time when the physician collected the fee and told the surgeon that the patient could pay nothing, or could pay but little, and either kept the whole or an undue portion, or took the divided fee and then rendered a separate bill. After this he became money-mad and sought subjects on which to satiate his sordid desires for easy gain. If the honest surgeon turned him down he sought those who would not and he has been busy ever since.

The surgeon who follows the lines of least resistance in these moral matters soon finds himself in the grasp of the system. Many very good, but quasi-respectable, men follow this practice, persuading themsedves that the doctor cannot be paid too much, and uphold the practice because it suits their convenience and is more profitable. Then, too, they say, "What's the difference?" The difference is most keenly realized when you have to tell your son, as I have mine, that our profession is becoming commercialized.

The prospects, therefore, are not encouraging for the profession, and we are compelled to admit that it is not what it once was. Its ideals are lowered. Its ethics may not be maintained and the best men under these conditions will not be eager to enter it.

Such courses, if pursued, will cost the profession its influence, cause loss of respect and confidence on the part of the people. Overcrowding and commercial competition will cheapen services and with the increase of endowed hospitals, dispensaries and laboratories, rewards for scientific medicine will be reduced. The general profession, which is already overshadowed by specialism will then be in hard lines. Every time a physician fails to make a proper and independent, self-respecting charge for the services rendered by him in the making of a diagnosis, recommending an operation, accompanying a patient to a hospital, being present, or assisting in an operation, he belittles himself, unduly aggrandizes the surgeon and discounts his own future usefulness, making it that much harder for those who follof him.

To be respected the physician must be self-respecting and respectable. He is the surgeon's friend and the surgeon will help him, if he wishes, to be independently, adequately and respectably paid for his valuable services to the patient and to the surgeon as well. Division of fees is wrong, dishonest and must be stopped for the good of all concerned. It has been my settled conviction for a long time that the physician is the ultimate loser by this practice, no matter how immediately profitable it may be to him. He is becoming dwarfed by his own shortsightednes and asphyxiated by a noose which was cast about him by a crafty specialist, perhaps, but which he himself is now tightening about his own throat.

There has been more harm done than can be overcome in a genration. Delayed action means more sacrifice. The organized profession should busy itself in reformation and take its stand in medical coleges, hospitals and socities, and as individuals and organizations, educate the public and reform our own members. The most hopeful view may be taken of the result. Any practice which even smacks of graft, or of obtaining money under false pretenses, cannot endure before a righteous public opinion. Nor will it continue in a profession which has cherished ideals from Hippocrates and the whole line of medical saints, religiously followed by the vast majority, during all the ages of the history of medicine.—*Jour. A. M. A., March* 11th, 1911.

---

Many, if not most, of the shoulder pain variously diagnosed as circumflex neuritis, reumatism, etc., are instances of subacromial or subcoracoid bursitis. With involvement of the former bursa there is usually tenderness just beyond the acromion, usually somewhat anteriorly; in the latter there is marked tenderness just external to the coracoid tip. Pain or rotation of the arm may be present in either; it is most constant in subcoracoid bursitis.—American Journal of Surgery.

